# Selection and Validation of Endogenous Reference Genes for qRT-PCR Analysis in Leafy Spurge (*Euphorbia esula*)

**DOI:** 10.1371/journal.pone.0042839

**Published:** 2012-08-14

**Authors:** Wun S. Chao, Münevver Doğramaci, Michael E. Foley, David P. Horvath, James V. Anderson

**Affiliations:** United States Department of Agriculture-Agricultural Research Service, Biosciences Research Lab, Sunflower and Plant Biology Research Unit, Fargo, North Dakota, United States of America; University of Michigan, United States of America

## Abstract

Quantitative real-time polymerase chain reaction (qRT-PCR) is the most important tool in measuring levels of gene expression due to its accuracy, specificity, and sensitivity. However, the accuracy of qRT-PCR analysis strongly depends on transcript normalization using stably expressed reference genes. The aim of this study was to find internal reference genes for qRT-PCR analysis in various experimental conditions for seed, adventitious underground bud, and other organs of leafy spurge. Eleven candidate reference genes (*BAM4*, *PU1*, *TRP-like*, *FRO1*, *ORE9*, *BAM1*, *SEU*, *ARF2*, *KAPP*, *ZTL*, and *MPK4*) were selected from among 171 genes based on expression stabilities during seed germination and bud growth. The other ten candidate reference genes were selected from three different sources: (1) 3 stably expressed leafy spurge genes (*60S*, *bZIP21*, and *MD-100*) identified from the analyses of leafy spurge microarray data; (2) 3 orthologs of *Arabidopsis* “general purpose” traditional reference genes (*GAPDH_1*, *GAPDH_2*, and *UBC*); and (3) 4 orthologs of *Arabidopsis* stably expressed genes (*UBC9*, *SAND*, *PTB*, and *F-box*) identified from Affymetrix ATH1 whole-genome GeneChip studies. The expression stabilities of these 21 genes were ranked based on the C_T_ values of 72 samples using four different computation programs including geNorm, Normfinder, BestKeeper, and the comparative ΔC_T_ method. Our analyses revealed *SAND*, *PTB*, *ORE9*, and *ARF2* to be the most appropriate reference genes for accurate normalization of gene expression data. Since *SAND* and *PTB* were obtained from 4 orthologs of *Arabidopsis*, while *ORE9* and *ARF2* were selected from 171 leafy spurge genes, it was more efficient to identify good reference genes from the orthologs of other plant species that were known to be stably expressed than that of randomly testing endogenous genes. Nevertheless, the two newly identified leafy spurge genes, *ORE9* and *ARF2*, can serve as orthologous candidates in the search for reference genes from other plant species.

## Introduction

Patterns of gene expression provide insight into the nature and behavior of genetic networks [1]. Quantitative real-time polymerase chain reaction (qRT-PCR) has become the most important tool in measuring levels of gene expression due to its accuracy, specificity, and sensitivity. The qRT-PCR technology is a great improvement over the original PCR methods developed by Mullis and coworkers [2]. It detects and quantifies the fluorescent signal after each amplification cycle; thus, combining amplification and detection into a single step in near real-time fashion. It also produces accurate data with a large dynamic range of at least 10^5^-fold [3] compared with 10^3^-fold in semi-quantitative PCR. The rate at which the fluorescent signal accumulates is directly dependent on the number of molecules of the target sequence in a given sample, and theoretically doubles during each round of amplification. During qRT-PCR, the cycle number at which the fluorescence generated within a reaction exceeds background level is referred to as the Cycle Threshold (C_T_). When comparing samples, the differences in C_T_ values are the log2 of the relative starting concentrations of the target cDNA. This method provides a very accurate measure of the differences in target cDNAs between samples, and thus is often used to examine changes in gene expression.

Although qRT-PCR offers clear advantages in RNA quantification, there are challenges associated with its use. These include difficulty in consistently maintaining equal quantities of starting materials, inherent variability in reverse transcription, and/or PCR efficiencies for RNA obtained from different tissues or tissue treatments, etc. [4], [5]. Therefore, good normalization methods are needed to compensate for sample-to-sample variation. Several strategies have been used for normalizing real-time PCR data [6], [7], [8], [9], [10]. For example, biological normalization uses identical sample amounts to extract RNA or uses an equal quantity of total RNA for reverse-transcription and real-time PCR reaction. Exogenous normalization uses a characterized RNA or DNA as a control and adds it into each sample at a known concentration. A passive reference dye, 6-carboxyl-X-rhodamine (ROX), is used to normalize non-PCR related factors affecting fluorescent signals including fluorescent fluctuations, well-to-well volume variations, and minor volume differences and changes in concentration [11]. Finally, genetic normalization uses endogenous reference genes such as glyceraldehyde-3-phosphate dehydrogenase, β-actin, 28 S and 18 S ribosomal RNA and many other stably-expressed genes to normalize RNA sample variation.

Among these normalization methods, endogenous reference genes are the most accepted and frequently used. An ideal reference gene should be stably expressed among samples, including those from different organs, developmental stages, and experimental conditions [12]. The reference gene is believed to compensate for any errors in the cDNA concentration for each sample incurred during cDNA preparation and/or PCR amplification [5], [8]. However, there is no single universal reference gene showing constant expression in all tissues. The commonly used “housekeeping genes” are no longer reliable sources for normalization of qRT-PCR data because their expression fluctuates substantially under different experimental conditions [13]. The use of such reference genes as normalizers could result in gross misinterpretations of many studies. The choice of a reference gene becomes particularly difficult when comparing different tissues and developmental stages since the transcriptomes differ strongly in those samples [14]. It is thus important to systematically validate the expression stability of candidate reference genes for transcript normalization. In addition, PCR amplification efficiency of the target and the reference genes can be very different, which also leads to significant biases and wrong data interpretation. For the above reasons, an extensive test is required to ensure that endogenous reference genes do express stably within the experimental settings.

Leafy spurge (*Euphorbia esula* L.) is an invasive weed that is estimated to cause significant economic losses annually in the Upper Great Plains of the USA [15]. This plant has a great ability to persist because of vegetative reproduction from adventitious crown and root buds and sexual reproduction through seeds. We have developed leafy spurge as an herbaceous perennial model to investigate transcriptome changes associated with dormancy responses in buds and seeds [16], [17]. In this work, we validated the expression stability of 21 candidate reference genes (Table 1 and Table S1) based on the analyses of qRT-PCR and four computational programs. Our approach was to find stably-expressed genes specifically suitable for normalization of transcripts during seed germination or bud growth. The expression levels of selected genes were then evaluated among other organs and experimental conditions. Eleven candidate reference genes were selected from 171 genes tested. We also examined 3 genes (*60S*, *bZIP21*, and *MD-100*) that exhibited stable expression based on leafy spurge microarray analyses. Moreover, we examined a few orthologs of *Arabidopsis* genes including 3 “general purpose” traditional reference genes (*GAPDH_1*, *GAPDH_2*, and *UBC*) and 4 stably expressed genes (*UBC9*, *SAND*, *PTB*, and *F-box*) that were identified from Affymetrix ATH1 whole-genome GeneChip studies [14]. Expression levels of each candidate reference gene were assessed by qRT-PCR using cDNAs prepared from 72 plant samples and the stability of gene expression was ranked using NormFinder, geNorm, BestKeeper, and Comparative ΔC_T_ software. Our results showed that some of these candidate reference genes outperformed the frequently used housekeeping genes. Among them, *SAND*, *PTB*, *ORE9*, and *ARF2* were chosen as good general purpose reference genes. The efficiency of PCR amplification was also determined for these potential reference genes using RNAs extracted from crown buds, meristems, and leaves.

**Table 1:** Abbreviations of genes mentioned in the manuscript.

Gene abbreviations	Gene names	*Arabidopsis* orthologue	Involved in
BAM4	Beta-amylase 4	At5g55700	Starch catabolic process
PU1	Pullulanase 1	At5g04360	Starch biosynthetic process
TPR-like	Tetratricopeptide Repeat-like	At4g39470	Protein–protein and protein–lipid interactions
FRO1	Frostbite 1	At5g67590	Cold acclimation
ORE9	Oresera 9, More Axillary Branches 2	At2g42620	Auxin polar transport and protein ubiquitination
BAM1	*E. esula* beta-amylase 1	At4g17090	Starch catabolic process
SEU	Seuss	At1g43850	Embryo and ovule development
ARF2	Auxin Response Factor 2	At5g62000	Floral organ abscission and leaf senescence
KAPP	Kinase Associated Protein Phosphatase	At5g19280	Signal transduction
ZTL	Zeitlupe	At5g57360	Regulation of circadian rhythm
MPK4	MAP Kinase 4	At4g01370	Signal transduction
MD-100	MD-100	Unknown	Unknown
60S	60S Ribosomal protein L18A	At2g34480	Ribosome biogenesis and translation
BZIP21	BZIP21 transcription factor	At1g08320	Regulation of transcription
GAPDH_1	Glyceraldehyde-3-phosphate dehydrogenase_1	At1g13440	Defense response and glycolysis
GAPDH_2	Glyceraldehyde 3-phosphate dehydrogenase_2	At1g13440	Defense response and glycolysis
UBC	Ubiquitin-Conjugating Enzyme	At5g25760	Fatty acid beta-oxidation and protein ubiquitination
UBC9	Ubiquitin-Conjugating Enzyme 9	At4g27960	Protein ubiquitination
SAND	SAND family protein	At2g28390	Vacuole fusion and endosomal traffic
PTB	Polypyrimidine Tract-Binding protein	At3g01150	Regulation of RNA splicing and translation
F-box	F-box domain	At5g15710	Unknown

## Results

### Identification of candidate reference genes

The goal of this research was to find internal reference genes for evaluating and normalizing transcript abundance in dormant and growth-induced seeds and adventitious underground buds of leafy spurge using qRT-PCR analysis. Three major sets of samples, germinating seeds (2007 and 2008), seasonally-harvested field buds (2003 and 2004), and growth-induced buds (2003 and 2004) were used. Gene expression was measured under different developmental stages for seeds and buds using qRT-PCR. A group of 109 genes was tested in seed samples, and another group of 62 genes was tested in bud samples. Genes that showed stable expression in one organ type (e.g., bud) were then tested in another organ type (e.g., seed). Differences in gene expression relative to their controls were evaluated and presented as ratios of log2 transformed relative expression values. Candidate reference genes were selected by their fold difference equal to or between −2 and 2 in log2 values (fold difference hereafter is based on log2 values) in sets of bud or seed experiments. July buds, 0 hr buds, and dry seeds were used as the control for seasonally-harvested field buds, growth-induced buds, and germinating seeds, respectively.

**Figure 1:**
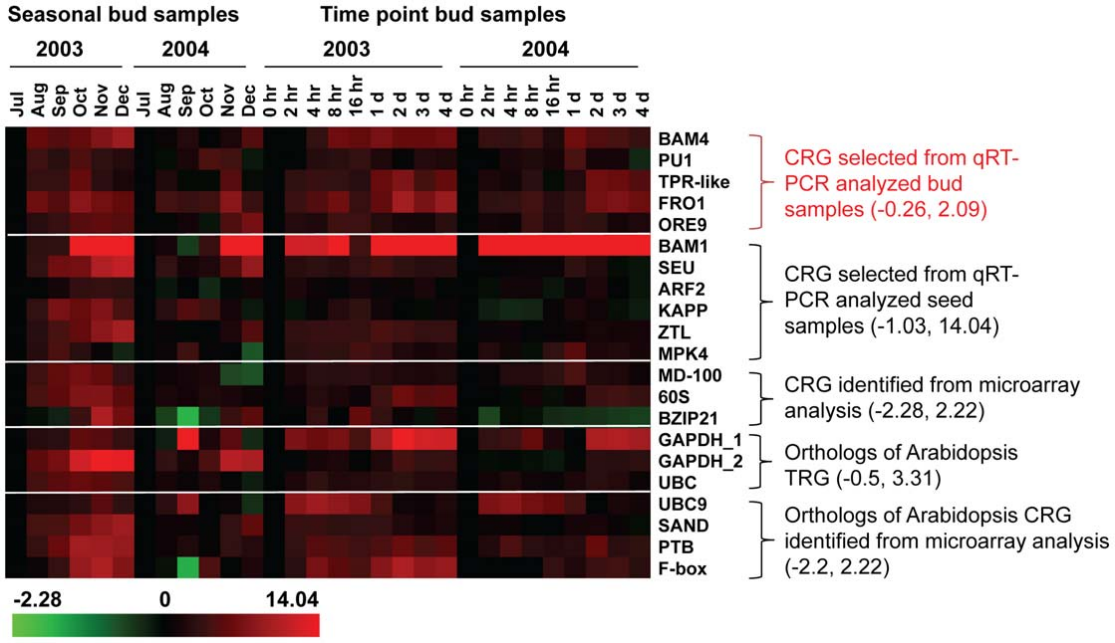
Candidate reference genes examined in bud samples. Two sets of seasonal bud samples (2003 and 2004) and two sets of time point bud samples (2003 and 2004) were used to examine gene expression. The fold difference is designated as log2 value. Red indicates up-regulated genes and green indicates down-regulated genes as compared with July or 0 hr buds (black). Bars at the bottom indicate the range of transcript changes in log2 value. The range of transcript changes is also shown inside the parenthesis. CRG: Candidate Reference Genes. TRG: Traditional Reference Genes.

**Figure 2:**
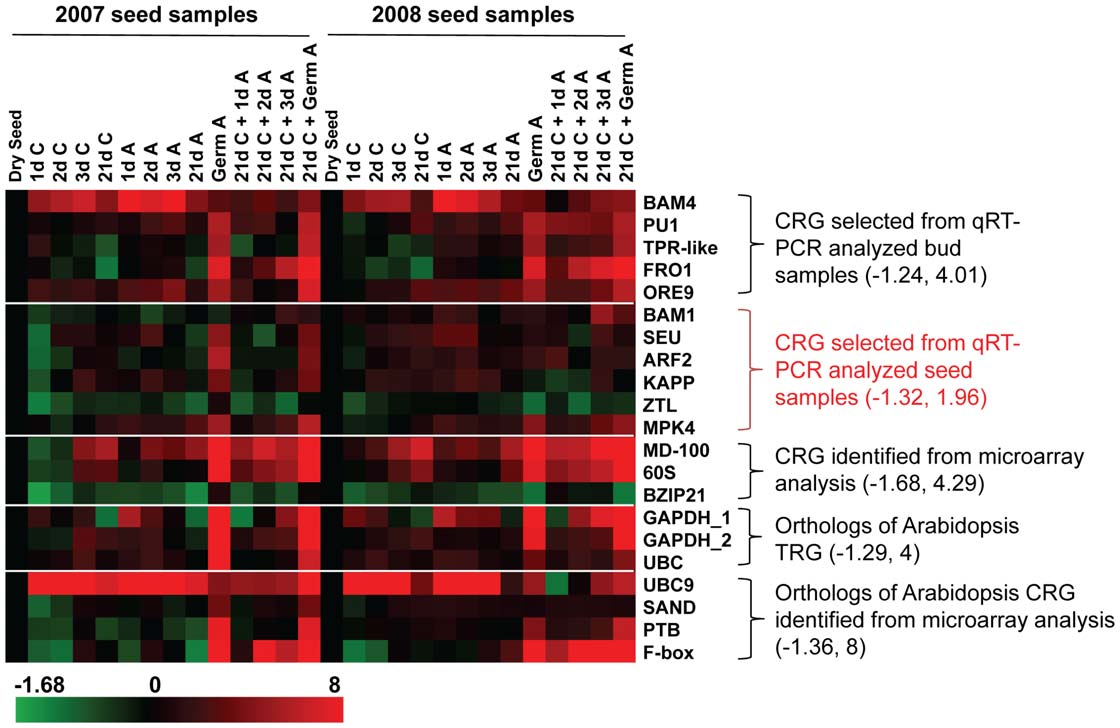
Candidate reference genes examined in seed samples. Two sets of germination treatment seed samples (2007 and 2008) were used to examine gene expression. The fold difference is designated as log2 value. Red indicates up-regulated genes and green indicates down-regulated genes as compared with dry seeds (black). Bars at the bottom indicate the range of transcript changes in log2 value. The range of transcript changes is also shown inside the parenthesis. CRG: Candidate Reference Genes. TRG: Traditional Reference Genes.

Among the 62 genes tested in bud samples, genes that showed a fold difference of −2 and 2 relative to July buds (for seasonal samples) or 0 hr buds (for time point samples) for both 2003 and 2004 were *BAM4*, *PU1*, *TRP-like*, *FRO1*, and *ORE9*; the expression range was −0.26 and 1.97 for seasonal samples and −0.43 and 2.09 for time point samples (Fig. 1 and Table S2). When these 5 genes were tested in seed samples (Fig. 2 and Table S3), the increase in gene expression for *PU1* and *ORE9* was around 2 for 2007 and 2008 samples (21d C+Germ A) relative to their controls. *BAM4* reached 2.7 for 2008 seeds (1d A). *FRO1* transcript was highly expressed in germinated seeds; the log2 value reached 4.0 for both 2007 and 2008 samples (21d C+Germ A). Among the 109 genes tested in seed samples, genes that showed a fold difference of −2 and 2 relative to dry seed were *BAM1*, *SEU*, *ARF2*, *KAPP*, *ZTL*, and *MPK4*; the expression range is −1.32 and 1.96 for 2007 sample, and −1.11 and 1.59 for 2008 sample (Fig. 2 and Table S3). When these 6 genes were tested in seasonal and time point bud samples, the log2 value for *ARF2*, *KAPP*, and *MPK4* were between −2 and 2 relative to their controls. However, the log2 value for *BAM1* increased to as high as 14 (2003 Dec buds) during seasonal progression relative to the July bud control (Fig. 1 and Table S2).

The three genes *MD-100*, *60S*, and *bZIP21* were identified as stably expressed based on leafy spurge microarray analyses. Our results showed that, although the expression of *MD-100* was quite stable in buds (Fig. 1), its log2 value differed as much as 4.29 between germinating (2007 Germ A) and dry seeds (Fig. 2 and Table S3). The expression levels of *60S* went up as high as 3.9 in germinated seeds (2007 Germ A and 21d C+Germ A) relative to dry seeds (Fig. 2 and Table S3). The gene, *bZIP21*, was stably expressed in seed samples with an expression range of −1.68 and 0.35 (Fig. 2 and Table S3) but it showed a relatively high expression range of −2.28 (2004, Sep) and 2.22 (2003, Nov) in buds relative to their July bud controls (Fig. 1 and Table S2). Orthologs of *Arabidopsis* traditional reference genes, *GAPDH_1*, *GAPDH_2*, and *UBC* showed divergent expression levels. Only *UBC* showed stable expression in bud samples ranging from −0.33 to 1.31 (Fig. 1 and Table S2). Both *GAPDH_1* and *GAPDH_2* were highly expressed in germinated seed samples, and the log2 value for *GAPDH_1* went as high as 4 in both 2007 and 2008 (21d C+Germ A) samples relative to dry seed controls (Fig. 2 and Table S3). In bud samples, *GAPDH_1* was highly expressed after decapitation and *GAPDH_2* was highly expressed during fall and winter (Fig. 1). Among the 4 *Arabidopsis* orthologs (*UBC9*, *SAND*, *PTB*, and *F-box*) identified as stably expressed genes from the whole-genome GeneChip studies [14], *SAND* is superlative and *PTB* rated second as reference gene candidates (Figs. 1 and 2). Both *UBC9* and *F-box* were unstably expressed in seeds; the expression of *F-box* increased by a log2 value as high as 8 (2007, Germ A) after seed germination relative to dry seeds (Fig. 2 and Table S3).

**Figure 3:**
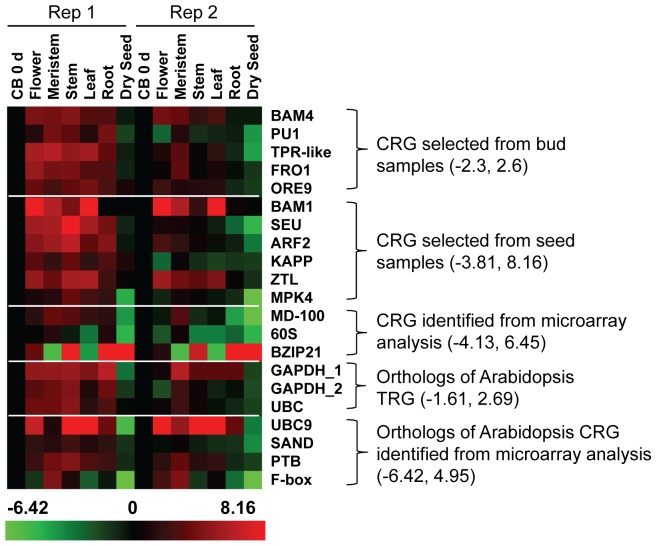
Candidate reference genes examined in different plant organs. Two sets of plant organ samples (Rep1 and Rep2) were used to examine gene expression. The fold difference is designated as log2 value. Red indicates up-regulated genes and green indicates down-regulated genes as compared with non-induced crown buds (CB 0 d, black). Bars at the bottom indicate the range of transcript changes in log2 value. The range of transcript changes is also shown inside the parenthesis. CRG: Candidate Reference Genes. TRG: Traditional Reference Genes.

The results shown above indicate that genes stably expressed in one organ type may not be stably expressed in another organ type. For example, *FRO1*, *MD-100*, *60S*, and *UBC9* were stably expressed in buds but very unstably expressed in seeds. Likewise, *BAM1* was stably expressed in seeds but extremely unstably expressed in buds. Thus, we also compared the expression of these 21 genes in various organ types including crown buds harvested from intact plants (CB 0 d), flowers, meristems, stems, leaves, roots, and dry seeds (Fig. 3 and Table S4). Genes that showed a fold difference equal to or between −2 and 2 relative to crown bud control for both replications were *BAM4*, *ORE9*, *KAPP*, *GADPH_2*, *UBC*, *SAND*, and *PTB*. Genes that showed a fold difference equal to or between −2 and 2.5 relative to crown bud control for both replications were *PU1*, *FRO1*, *ARF2*, and *ZTL*. The rest of genes were outside the range of −2 and 2.5 relative to crown bud controls. Among them, the expression of *BAM1*, *BZIP21*, *UBC9*, and *F-box* were considered to be extremely unstable.

### Expression levels and ranking of candidate reference genes

#### Average cycle threshold (C_T_) values of candidate reference genes

**Figure 4:**
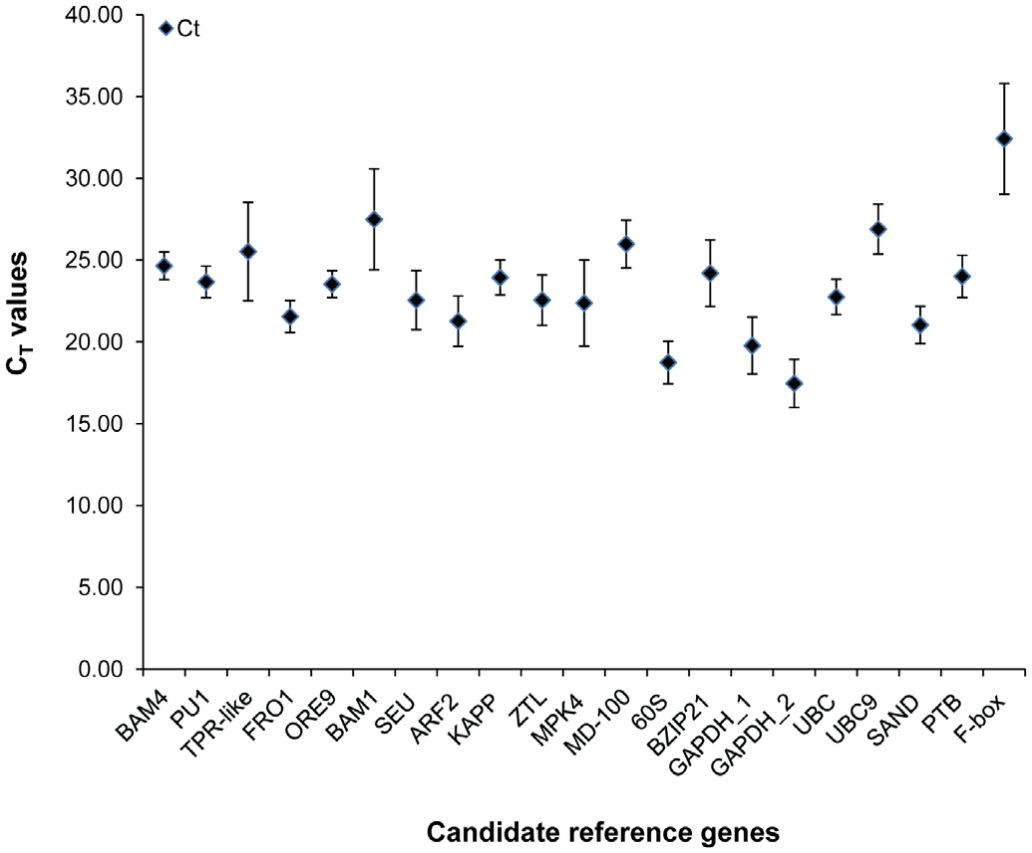
Average cycle threshold (C_T_) values for 21 candidate reference genes. The filled diamond symbol indicates median C_T_ values. The bars indicate standard deviation.

The cycle threshold (C_T_) value is the amplification cycle number at which the fluorescence rises above the threshold setting. Since all qRT-PCR reactions were performed with an equivalent amount of template cDNA, transcript abundance of these genes in different samples may be estimated by direct comparison of C_T_ values (Fig. 4 and Table S5). Figure 4 shows the median C_T_ values of 21 candidate reference genes after averaging the C_T_ values of 72 different samples including buds, seeds, and various organs (see Table S5 for C_T_ values). Most of the genes displayed median C_T_ values ranging from 20 to 25, which is considered a moderate to high level of expression. The genes *60S* and *GAPDH_2* showed relatively high expression with median C_T_ values ranging from 15 to 20, and *BAM1*, *MD-100*, and *UBC9* showed relatively low expression with median C_T_ values ranging from 25 to 30. *F-box* (mean C_T_ 32.42) was expressed at the lowest level. Standard deviation of C_T_ values can reveal the expression stability of candidate reference genes. For example, the expression of *TPR-like*, *BAM1*, and *F-box* genes are highly variable as evidenced by their large standard deviations and the expression of *BAM4*, *PU1*, and *ORE9* are relatively stable with small standard deviations. However, more robust stability ranking was obtained using the four different computational programs shown below.

#### Stability ranking of candidate reference genes

The stabilities of the 21 candidate reference genes were ranked within buds (30 samples), seeds (28 samples), among different organs (14 samples), and among all samples which included the aforementioned three categories: buds, seeds, and organs (72 samples). The C_T_ values (Table S5) for each candidate reference gene were used for stability comparison in the NormFinder, geNorm, BestKeeper, and Comparative ΔC_T_ programs to identify the best reference genes for qRT-PCR data normalization in biological samples. The results of the analyses for top 10 genes are given in Table 2 (see also Table S6 for ranking results of all 21 genes). In bud samples (Table 2A), 6 genes (*ZTL*, *ARF2*, *SEU*, *SAND*, *PTB*, and *MPK4*) were identified among the top 3 reference genes in each of the 4 computational programs. The overall ranking of the best reference genes (using Recommended Comprehensive Ranking method) for bud was *SAND*, *ZTL*, and *SEU*. In contrast, the overall ranking of the worst reference genes (using Recommended Comprehensive Ranking method) for buds was *TRP-like*, *BAM1*, and *GAPDH_1* (Table S6A). Likewise, in seed samples, 8 genes (*MPK4*, *BAM1*, *UBC*, *ARF2*, *SAND*, *ZTL*, *PTB*, and *BZIP21*) were identified (Table 2B), and based on the Recommended Comprehensive Ranking method the 3 best were *SAND*, *ARF2*, and *UBC* and the 3 worst were *F-box*, *UBC9*, and *GAPDH_1* (Table S6B). In organ samples, 7 genes (*PTB*, *KAPP*, *ARF2*, *BAM4*, *UBC*, *SAND*, and *ORE9*) were identified (Table 2C), and based on the Recommended Comprehensive Ranking method the 3 best were *PTB*, *UBC*, and *SAND* and the 3 worst were *BZIP21*, *BAM1*, and *F-box* (Table S6C). When including the data obtained from all the samples (buds, seeds, and organs) into the analysis, we identified 6 genes (*SAND*, *ORE9*, *UBC*, *PTB*, *BAM4*, and *FRO1*) of which *SAND*, *PTB*, and *UBC* were identified as the best reference candidates (Table 2D) and *TRP-like*, *BZIP21*, and *BAM1* as the worst (Table S6D).

**Table 2:** Stability ranking of candidate reference genes.

Methods	A. Bud Ranking Order (Better–Good–Average)									
	1	2	3	4	5	6	7	8	9	10
Delta CT	ZTL	PTB	SAND	ORE9	UBC	SEU	ARF2	60S	KAPP	FRO1
BestKeeper	ARF2	MPK4	SAND	ORE9	KAPP	60S	ZTL	SEU	UBC	PTB
Normfinder	SEU	ZTL	SAND	PTB	GAPDH_2	ORE9	60S	ARF2	FRO1	UBC
Genorm	SEU | SAND	ZTL	ORE9	ARF2	PTB	UBC	60S	KAPP	FRO1	
Recommended comprehensive ranking	SAND	ZTL	SEU	ARF2	ORE9	PTB	60S	UBC	KAPP	MPK4

	B. Seed Ranking Order (Better–Good–Average									
Delta CT	MPK4	UBC	SAND	ARF2	PTB	ORE9	PU1	TPR-like	KAPP	SEU
BestKeeper	BAM1	ZTL	SAND	ARF2	BZIP21	KAPP	UBC	SEU	MPK4	ORE9
Normfinder	UBC	MPK4	PTB	ORE9	PU1	ARF2	SAND	TPR-like	KAPP	GAPDH_2
Genorm	ARF2 | SAND	BZIP21	ZTL	KAPP	SEU	UBC	MPK4	ORE9	PU1	
Recommended comprehensive ranking	SAND	ARF2	UBC	MPK4	BAM1	ZTL	ORE9	PTB	KAPP	BZIP21

	C. Organs Ranking Order (Better–Good–Average)									
Delta CT	PTB	UBC	ARF2	SAND	FRO1	BAM4	TPR-like	ORE9	SEU	GAPDH_2
BestKeeper	KAPP	SAND	ORE9	UBC	GAPDH_2	FRO1	PU1	PTB	BAM4	ARF2
Normfinder	ARF2	PTB	BAM4	SAND	UBC	FRO1	ORE9	TPR-like	SEU	ZTL
Genorm	BAM4 | PTB	UBC	FRO1	SAND	ORE9	ARF2	ZTL	TPR-like	SEU	
Recommended comprehensive ranking	PTB	UBC	SAND	BAM4	ARF2	FRO1	ORE9	KAPP	GAPDH_2	TPR-like

	D. All samples Ranking Order (Better–Good–Average)									
Delta CT	SAND	PTB	UBC	PU1	ORE9	ARF2	GAPDH_2	KAPP	60S	ZTL
BestKeeper	ORE9	BAM4	FRO1	PU1	KAPP	UBC	SAND	60S	PTB	UBC9
Normfinder	SAND	PTB	UBC	ORE9	GAPDH_2	PU1	60S	KAPP	ARF2	ZTL
Genorm	UBC | PTB	SAND	KAPP	ARF2	GAPDH_2	PU1	60S	MD-100	ORE9	
Recommended comprehensive ranking	SAND	PTB	UBC	ORE9	PU1	KAPP	GAPDH_2	ARF2	60S	FRO1

In Genome calculations, the top 2 genes cannot be resolved. A full list of 21 genes is in Table S6.

### Determine the efficiency of PCR amplification

PCR amplification efficiency is defined as copies (exponential amplification) or percentage (efficiency) of PCR product increase per cycle. Similar amplification efficiency between reference and target genes is recommended for reliable comparison between samples, especially when comparison is performed based on the relative quantification method [23]. Therefore, amplification efficiencies of the top 10 reference genes were determined (Table S7). These 10 genes were chosen from the overall ranking of the best reference genes for all samples (see Table 2D), since ideal reference genes should be applicable to all samples. Amplification efficiencies were determined using RNA samples prepared from crown buds, meristems, and leaves. Among 30 averaging efficiency values (10 genes in 3 different tissues), 5 were between 90% and 99%, 18 were between 80% and 89%, and 7 were between 70% and 79%. The results showed that the differences in amplification efficiencies were within 5% between two biological reps for most samples; only ORE9 and GAPDH_2 were found between 5% and 10% in leaf samples. However, the efficiency values were not necessarily similar among the three different organs tested. For example, over half of the genes exhibited a difference between 5% and 10% and *PTB* and *UBC* produced efficiency differences over 10%. Since amplification efficiency can be very diverse in different tissues, it may be necessary to incorporate a correction for amplification efficiency into the analysis in gene expression studies [28].

## Discussion

The expression stability of 21 genes was tested in 72 RNA samples including various organs and different developmental stages of buds and seeds from two biological replicates. Our study showed that the status of seeds significantly affected the expression level of these genes; most of the genes were up-regulated during growth (Fig. 2, Germ A and 21d C+Germ A), similar to a phenomenon observed previously [20]. Therefore, it is extremely important to select reference genes that are not affected by germination and growth. Genes were also identified that were very stably expressed during seed germination but were extremely variable in bud growth. For example, the expression range for *BAM1* was −0.7 and 1.59 (in log2 value) in seeds and was −0.73 and 14.05 in buds (Figs. 1 and 2, Tables S2 and S3). Based on these results, a preliminary analysis of the stability of reference genes is highly recommended before conducting a gene expression analysis by qRT-PCR in new experimental settings.

Expression stabilities of these 21 genes were ranked in buds, seeds, organs, and all samples using the four different computation programs. Our data showed that these 4 computational programs did not place the order of top ranked genes equally. This discrepancy implies differences in the statistical algorithms. However, the top 3 genes evaluated from one program were, in general, within the top 10 genes from the analyses of the other 3 programs. These 4 programs rank unsuitable reference genes more consistently perhaps due to their wide variability in gene expression. Since ideal reference genes are considered to be stably expressed in different organs at various developmental stages, the following discussion is primarily based on the results of overall ranking of the best reference genes for all samples (Table 2D). The ranking order from better to average is as follows: *SAND*, *PTB*, *UBC*, *ORE9*, *PU1*, *KAPP*, *GADPH_2*, *ARF2*, *60S*, and *FRO1*.

The *SAND* gene not only ranked first among 21 genes from the results of overall ranking of the best reference genes for all samples, but also first from the results of overall ranking of the best reference genes for buds and seeds (Table 2 A and B).The SAND family protein is involved in vacuole fusion at the tethering/docking stage in yeast [29] and endosomal traffic in *Caenorhabditis elegans* [30]. We tested *SAND* because it was one of the stably expressed *Arabidopsis* genes identified from Affymetrix ATH1 whole-genome GeneChip studies [14]. This *SAND* was also one of the most stably expressed genes in *Arabidopsis* after exposure to Cd and Cu [31] and in different tissues, organs, and pathogen challenged leaves in citrus [32]. The *F-box* gene, on the other hand, was also identified from Affymetrix GeneChip studies and was among one of the highest stably expressed genes in *Arabidopsis* and citrus from the aforementioned studies; however, this gene was quite unstable during seed germination (Fig. 2 and Table S3), indicating that levels of gene expression are sometimes species-specific. The *PTB* gene was also identified from the Affymetrix GeneChip studies and was ranked second in all sample analysis. It encodes a RNA-binding protein that binds pre-mRNAs and regulates alternative pre-mRNA splicing [33]. Although this gene was regarded as highly stable based on the analysis of the four computation programs, *PTB* expression was modestly increased in germinated seeds (log2 went as high as 2.53, Fig. 2 and Table S3) and thus may not be an ideal reference gene when studying seed germination.

The *UBC* gene was ranked the third among 21 genes from the results of overall ranking of the best reference genes for all samples. This *UBC* gene encodes an ubiquitin-conjugating enzyme (also known as E2 enzyme) that is involved in protein degradation through ubiquitination reactions. This gene performed best among the three traditional housekeeping genes tested; however, since *UBC* exhibited two peaks in dissociation analysis, it is thus not considered a good reference gene. The 4^th^ ranking gene *ORE9* (also called *MAX2*) encodes an F-box protein that regulates leaf senescence and controls shoot lateral branching in *Arabidopsis* [34], [35]. The *ORE9* transcript was modestly increased in germinated seeds; however, the levels of expression appeared more stable than those of *PTB* (Fig. 2 and Table S3). The genes *PU1*, *KAPP*, and *GADPH_2* were ranked in positions 5, 6, and 7, respectively, according to overall ranking of the best reference genes for all samples. However, they were ranked equal to or behind the 8^th^ position from the results of overall ranking of the best reference genes for buds, seeds, and organs (Table 2 A, B, and C); thus, they are not considered as suitable references genes. The 8^th^ ranking gene *ARF2*, on the other hand, was ranked 4, 2, and 5 according to the overall ranking of the best reference genes for buds, seeds, and organs, respectively (Table 2 A, B, and C), and thus is considered a good reference gene. The *ARF2* gene encodes an auxin response transcription factor that regulates leaf senescence in *Arabidopsis* [36].

In summary, *SAND*, *PTB*, *ORE9*, and *ARF2* seemed to be the most appropriate general purpose reference genes for accurate normalization of gene expression data. Among these 4 genes, *SAND* and *PTB* were orthologs of *Arabidopsis* described as stably expressed based on Affymetrix ATH1 whole-genome GeneChip studies. The other 2 genes, *ORE9* and *ARF2*, were selected directly from the expression studies of 171 leafy spurge genes. Our results showed that it was more efficient to identify good reference genes from the orthologs of other plant species that were known to be stably expressed than that of randomly testing endogenous genes. Nevertheless, these newly identified leafy spurge genes can serve as orthologous candidates in searching of reference genes for other plant species. Our results also showed that the levels of transcripts from the traditional housekeeping genes *GAPDH_1* and *GAPDH_2* were very unstable in buds, seeds, and various organs, indicating again the importance of validating these housekeeping genes before using them for normalization purposes. Furthermore, it is well known that using a single reference gene cannot adequately normalize sample variations and the geometric mean of multiple reference genes provides much better normalization results [25]. Identification of different reference genes provides flexibility in combinations of two or more genes to best normalize leafy spurge qRT-PCR data.

## Materials and Methods

### Preparation of seed samples

Field-grown leafy spurge seeds were collected from Fargo, ND USA in 2007 and 2008. The seed procurement and handling methods have been previously described [18]. Seeds for each treatment were surface disinfected for 10 min with a 50% (v/v) solution of commercial bleach (6.25% NaOCl) containing a drop of Triton X-100 surfactant and rinsed 10 times for 1 to 2 min with sterile distilled water. Fourteen treatments including (a) dry seed, (b) 1d C, (c) 2d C, (d) 3d C, (e) 21d C, (f) 1d A, (g) 2d A, (h) 3d A, (i) 21d A, (j) Germ A, (k) 21d C+1d A, (l) 21d C+2d A, (m) 21d C+3d A, and (n) 21d C+Germ were examined to study gene expression during seed dormancy and growth. The surface-disinfected seeds were re-dried in the laminar flow hood for about 2 h to their original fresh weight, and this is designated as treatment a, dry seed. For treatments b, c, d, and e, seeds were incubated at 20°C, respectively for 1, 2, 3, and 21 d. For treatments f, g, h, and i, seeds were incubated at the alternating temperature of 20∶30°C (16∶8 h), respectively for 1, 2, 3, and 21 d. Treatment j was germinated seeds that were incubated for 2 to 21 d at the alternating temperature. For treatments k, l, and m, seeds were incubated for 21 d at 20°C followed by 1, 2, and 3 d, respectively at the alternating temperature. Treatment n was germinated seeds that were incubated for 21 d at 20°C followed by alternating temperature for 2 to 21 d. All experiments were done in Petri dishes and kept in the dark, except for short period of rating and harvesting germinated seeds. After treatment, seeds were frozen in liquid nitrogen and maintained at −80°C until extraction of RNA. Germinated seeds, as defined by the first sign of testa rupture, were collected and immediately frozen in liquid nitrogen over the 21 d period to obtain a sufficient sample of seeds. Transcriptome profiles were very different among these treatments as evidenced by previous publications [19], [20]. A total of 28 individual seed samples including two biological replicates were used to examine the expression of genes using qRT-PCR.

### Preparation of crown bud samples

Greenhouse- and field-grown leafy spurge plants were used for crown bud sample preparation. Greenhouse plants were started as shoot cuttings from Biotype 1984-ND-001 and maintained by clonal propagation. Shoot cuttings from greenhouse-grown plants were placed in Sunshine #1 potting mix (Fisons Horticulture Inc., Bellevue, WA) inside 4×21 cm Ray Leach Cone-tainers (SC-10 super cell, Stuewe and Sons Inc., Corvallis, OR) and grown in a greenhouse under a 16∶8 h day∶night photoperiod cycle at 28±4°C for 3–4 mo. Growth-induced crown bud samples were harvested from greenhouse-grown leafy spurge. These crown buds were harvested 0 h, 2 h, 4 h, 8 h, 16 h, 1 d, 2 d, 3 d, and 4 d after shoot removal. Two sets of growth-induced crown buds were harvested from 2003 and 2004. Seasonal bud samples were harvested from field-grown leafy spurge plants. Field-grown plants were established by transplanting a portion of the greenhouse population to a field plot and seasonal buds were harvested monthly from July to December of 2003 and 2004. A total of 30 individual bud samples including 2 sets of seasonal (2003 and 2004) and 2 sets of growth-induced (2003 and 2004) samples were used to examine the expression of genes using qRT-PCR.

### Preparation of various plant organs

Leafy Spurge organs were harvested directly into liquid nitrogen from four month old greenhouse-grown plants in June and August, 2011, unless otherwise noted. Meristems were harvested by peeling back the young unemerged leaves surrounding the terminal meristem. Stems and leaves were isolated from the top quarter of the plants. Roots and crown buds were rinsed free of all soil and then removed from the plant. The same 2007 and 2008 dry seed (see above) were used for this study. Whole flowers were harvested from the field-grown plants and immediately placed into liquid nitrogen at two different time points, June and August, 2011. A total of 14 individual organ samples including two biological replicates were used to examine the expression of genes using qRT-PCR.

### cDNA template preparation and quantitative Real-Time PCR (qRT-PCR)

Total RNAs were extracted from different tissues using the pine tree extraction protocol [21] and used to prepare cDNA template through reverse transcription according to manufacturer's instructions (Invitrogen). Briefly, 5 µg of total RNA was DNase treated and then reverse transcription was performed in 20 µl total volume using a SuperScript First-Strand Synthesis Kit to produce total cDNA from each sample. After cDNA synthesis, each 20 µl reaction was diluted to 800 µl and stored at −80°C.

Gene expression by qRT-PCR was examined using cDNA templates on an Applied Biosystems 7300 Real-Time PCR System. For real-time PCR reactions, 2 µl total cDNA was added to a 20 µl PCR reaction mixture containing 10 µl of 2× Power SYBR Green PCR Master Mix and 0.5 µl of each primer (20 pmol). Thermal cycling was performed with a Thermal Profile step of 2 min at 50°C, Auto Increment step of 10 min at 95°C, and followed by 40 cycles of 20 s at 95°C, 10 s at various annealing temperatures (50–58°C), and 35 s at 72°C. For dissociation analysis, a temperature ramp step was added to the end of the thermal profile with an initial temperature of 55°C and a final temperature of 95°C. Polymerase chain reactions were electrophoresed on 1% agarose gels. Primers (20–24 nucleotides) were designed using Lasergene sequence analysis software (DNASTAR, Inc., Madison, WI) from clones annotated to genes based on sequences obtained from a leafy spurge EST-database [16]. Primer sequences and qRT-PCR conditions are shown in Table S1.

The comparative C_T_ method was used to determine changes in target gene expression in test samples relative to a control sample. The formula used to calculate the fold differences is similar to the standard comparative C_T_ method (ΔΔC_T_) except that no endogenous reference gene is incorporated in the calculation since we want to determine stably expressed genes before normalization. The modified formula for fold difference in gene expression of test vs control sample is ΔC_T_ = ΔC_T,test_−ΔC_T,control_. Here, ΔC_T,test_ is the C_T_ value of the test sample, and ΔC_T,control_ is the C_T_ value of the control sample. The chemistry of SYBR green was used to produce fluorescent signals and two technical replicates were used per sample for the qRT-PCR experiments. The C_T_ value of each gene is the average of its two technical replicates. The 2007 and 2008 seed, 2003 and 2004 bud, and 2011 June and August organ samples served as the two biological replicates for each set of tissues. Passive reference dye, ROX, was used to normalize for non-PCR-related fluctuations in fluorescent signals. The difference in gene expression is designated as log2 value. Heat-maps of the qRT-PCR results were created based on log2 values using Eisen Lab software, Cluster and TreeView as described by Eisen et al. [22].

To determine PCR amplification efficiency, a 5-fold serial dilution of the template cDNA was made, and the log concentration of the template vs C_T_ was then plotted using Excel with the log input amount as the X value and C_T_ as the Y value [23]. The slope of the trend line is a function of the PCR amplification efficiency. The formula for exponential amplification (copies per cycle per template DNA) is 10^(−1/slope)^, and the formula for efficiency is 10^(−1/slope)^−1.

### Ranking the stabilities of candidate reference genes

RefFinder [24] was used to determine the stabilities of candidate reference genes. RefFinder is a web-based tool that integrates the current major computational programs, including geNorm, Normfinder, BestKeeper, and the comparative ΔC_T_ method, to compare and rank the stability of candidate reference genes. The C_T_ value for each candidate reference gene was used by these programs to determine its relative expression stability. Among these four programs, geNorm, Normfinder, and BestKeeper are Excel-based software tools. Also, NormFinder and geNorm use relative expression values as input data, whereas BestKeeper and the comparative ΔC_T_ method use C_T_ values directly.

The geNorm program provides the two most stable reference genes or a combination of multiple stable genes by calculating a gene expression normalization factor (M value) based on the geometric mean of a number of candidate reference genes [25]. NormFinder identifies the optimal reference gene among a group of candidate genes based on their expression stability in a sample set or specific experimental designs [26]. This algorithm evaluates the overall expression variation of the candidate reference genes and the variation between subgroups of samples. BestKeeper determines the best reference genes using pair-wise correlation analysis of candidate reference genes [4]. BestKeeper uses standard deviation, percent covariance, and power of the candidates as indicators to determine the best reference genes. The comparative ΔC_T_ method evaluates the most stable reference genes by comparing relative expression of “pairs of genes” within each sample [27]. This method measures the stability of a gene by the mean of standard deviation values derived from comparison between a reference gene and other candidate reference genes. Overall ranking of the best reference gene is obtained using the ranking results of all four algorithms. The detailed calculation procedures are described in Chen et al. [24].

## Supporting Information

Table S1 **Abbreviations of genes and primer sequences.**
(XLSX)

Table S2 **Candidate reference genes examined in bud samples.**
(XLSX)

Table S3 **Candidate reference genes examined in seed samples.**
(XLSX)

Table S4 **Candidate reference genes examined in different plant organs.**
(XLSX)

Table S5 **Cycle threshold (C_T_) values for 21 candidate reference genes.**
(XLSX)

Table S6 **Stability ranking of candidate reference genes.**
(XLSX)

Table S7 **Amplification efficiencies of the top 10 reference genes.**
(DOCX)
