# Supplementary material for: Selection and Validation of Endogenous Reference Genes for qRT-PCR Analysis in Leafy Spurge (Euphorbia esula)
Source: PLoS One. 2012 Aug 14;7(8):e42839. doi: 10.1371/journal.pone.0042839 (PMC3419244; doi:10.1371/journal.pone.0042839)
Supplement: Table S7 — Amplification efficiencies of the top 10 reference genes. (DOCX) [file pone.0042839.s007.docx]

Supplementary Table 7. Amplification efficiencies of the top 10 reference genes

| Gene | Crown bud | | | Meristem | | | Leaf | | |
| --- | --- | --- | --- | --- | --- | --- | --- | --- | --- |
|  | Rep1 | Rep2 | Average | Rep1 | Rep2 | Average | Rep1 | Rep2 | Average |
| SAND | 83 | 82 | 83 | 86 | 85 | 86 | 90 | 88 | 89 |
| PTB | 89 | 86 | 88 | 80 | 76 | 78 | 90 | 92 | 91 |
| UBC | 83 | 82 | 83 | 72 | 75 | 74 | 68 | 73 | 71 |
| ORE9 | 87 | 83 | 85 | 86 | 83 | 85 | 76 | 85 | 81 |
| PU1 | 88 | 86 | 87 | 90 | 90 | 90 | 86 | 90 | 88 |
| KAPP | 84 | 84 | 84 | 92 | 88 | 90 | 92 | 90 | 91 |
| GAPDH_2 | 89 | 92 | 91 | 82 | 84 | 83 | 85 | 91 | 88 |
| ARF2 | 82 | 79 | 81 | 84 | 82 | 83 | 80 | 83 | 82 |
| 60S | 75 | 70 | 73 | 72 | 71 | 72 | 74 | 76 | 75 |
| FRO1 | 83 | 83 | 83 | 76 | 78 | 77 | 80 | 80 | 80 |
